# Subcutaneous dirofilariosis (*Dirofilaria repens*): an infection spreading throughout the old world

**DOI:** 10.1186/s13071-017-2434-8

**Published:** 2017-11-09

**Authors:** Claudio Genchi, Laura Kramer

**Affiliations:** 1 Dipartimento di Medicina Veterinaria, Università degli Studi di Milano, 20133 Milan, Italy; 2 Dipartimento di Scienze Medico Veterinarie, Università di Parma, Parma, Italy

**Keywords:** *Dirofilaria repens*, Subcutaneous dirofilariosis, Zoonosis

## Abstract

**Background:**

Two main *Dirofilaria* species infect dogs: *D. immitis* and *D. repens*. While *D. immitis* has a worldwide distribution, *D. repens* is currently found only in Europe, Asia, and Africa. Adult *D. repens* are located in subcutaneous tissues of natural hosts where they survive for long periods of time. First-stage larvae, microfilariae, circulate in the peripheral bloodstream, where they are taken up by the mosquito intermediate hosts. Infected mosquitoes then transmit infective third-stage (L3) larvae to new hosts through the blood meal. In dogs, most infections are asymptomatic, although cutaneous disorders such as pruritus, dermal swelling, subcutaneous nodules, and ocular conjunctivitis can be observed. Currently, two factors have increased the concerns about this parasitic infection 1) its spread throughout the European countries and to other continents and its prevalence in dog populations, where in some cases it has overcome *D. immitis;* and 2) its zoonotic potential, which is much greater than that of *D. immitis*.

**Results:**

Different hypotheses can be put forward to explain these concerns. First, climate change has allowed more favorable conditions for survival of culicid vectors. Second, accidental hosts such as humans may have a less efficient immune reaction against a parasite that is located in subcutaneous tissues, and thus less exposed to the host’s immune response than, for instance, *D. immitis*. Furthermore, the absence of clinical signs in the majority of canine infections and the difficulty in diagnosing the infection, due to the lack of serologic tests and thus the reliance on the identification of microfilariae and differentiation from *D. immitis* to confirm the presence of the parasite, favor the further spread of this species. Finally, among the macrocyclic lactones currently used to prevent heartworm infection, only moxidectin has been found to be fully effective against the infective larvae transmitted by mosquitoes and partially effective (efficacy 96%) against adult *D. repens* in experimental studies.

**Conclusions:**

*Dirofilaria repens* infection is much more difficult than *D. immitis* to diagnose and control in the reservoir population (microfilaremic dogs). In addition, lack of familiarity with *D. repens* infection could lead to lack of vigilance underestimation for this parasite*.* The number of human cases in Europe and Asia is currently a serious public health concern. Medical doctors and veterinarians must collaborate closely for better control and surveillance of *D. repens* infection.

## Background

Dirofilarial infections are vector-borne parasitic diseases mainly of dogs, cats, and wild carnivores caused by *Dirofilaria* (*Noctiella*) *repens* and *D. immitis. D. repens* is endemic in many countries of the Old World but has not yet been found in the Americas (reports from Brazil and Peru [1, 2] are doubtful and need confirmation), while *D. immitis* infections have a worldwide distribution [3]. The two parasites were confused for many years and it was assumed that *D. immitis* worms could be found both in the pulmonary artery and in subcutaneous tissues. In 1910, Bonvicini [4], an Italian scientist, while studying the effect of essential turpentine oil against what he supposed were heartworms, recovered some adult worms from subcutaneous tissues of a necropsied dog and sent them to Raillet and Henry in Lyon (France), who subsequently described and named the species *D. repens* [5]. Nonetheless, the first observation of *D. repens* was probably from a human being. In 1566, a Portuguese medical doctor, Amato Lusitano, reported in his *Curationum Medicinalium Centuriae*: “*puella trima … per oculi internam partem, quam angulum magnum appellamus, a jumbrici cuiusdamcaput appere coepis…*” (in a three-year-old girl, suddenly it started to appear in the area we call big angle of the eye the tip of one worm which sometimes are sited in the eye making its opacity) [6].

*D. repens* has a long pre-patent period (170–238 days) [7], although in a recent experimental study the first microfilariae were found in the bloodstream on day 164 post infection [8]. Infective larvae migrate throughout the subcutaneous tissue and muscular connective fasciae, where they develop to the adult stage and reside permanently. Adult worms and microfilariae are long-living parasites in their natural hosts (about 4 years). The parasite, like other filarioids*,* harbors an endosymbiont bacterium (*Wolbachia*) that plays a significant role in desensitization of host innate immunity, assuring the worms’ long-term survival [9]. Interestingly, no inflammatory reaction or connective tissue capsule surrounds the living parasite, which can be seen moving actively under the connective serous layers [8, 10]. In most cases, including experimental infections, the infection goes unnoticed (no clinical signs) [8, 10]. Occasionally, cutaneous disorders such as pruritus, dermal swelling, subcutaneous nodules containing the parasite, and ocular conjunctivitis can be observed [11]. Allergic reactions likely due to microfilaria sensitization and *Wolbachia*-mediated inflammatory reactions in severe infections have been reported [12]. In these cases, circulating microfilariae often are absent.

### Canine infections

Infected dogs are the main reservoir of *D. repens* infection as they frequently have microfilariae in the peripheral blood. Since the early 1900s, canine *D. repens* infection has been reported as endemic in central and southern Italy and in other southern European countries, such as France, Greece, and the former Yugoslavia [13]. Several factors have contributed to the spread of canine *D. repens* infection. The introduction of the Pet Travel Scheme in 2000 allowed easier movement of companion animals throughout the European Union [14]. Rising temperatures have resulted in the spread throughout Europe of the Asian tiger mosquito (*Aedes albopictus*) and other competent vector species such as *Ae. koreicus* [15]. Currently new endemic areas have been identified and confirmed in Austria, Czech Republic, Germany, Hungary, Poland, Russia, Ukraine, Slovakia, Turkey, and the Balkan Peninsula (Albania, Bosnia, Bulgaria, Croatia, Greece, Macedonia, Romania, Serbia) [16–19]. It is noteworthy that in several eastern European countries *D. repens* prevalence is higher than *D*. *immitis*: for example, in Romania, where the heartworm prevalence ranges 02% to 2% and the *D. repens* prevalence range is from 7% to 20% [20–22].

### Human infections

Humans can be accidental hosts for both *D. immitis* and *D. repens*. Comparing the data of zoonotic infections caused by *D. immitis* in the United States and Japan with that observed in Europe in recent years, however, the figures are surprisingly different. Approximately 110 human cases of *D. immitis* have been reported in the US in the last 50 years [23, 24] and 277 cases in the last 39 years in Japan [25], while in Europe about 25 cases have been reported in the last 37 years [26–28]. It is noteworthy that the prevalence of canine heartworm infection in endemic areas of the US, Japan, and Europe is essentially the same (12% to 60% in dogs not given preventives). For *D. repens*, more than 3500 human cases were reported in Europe from 1977 to 2016 [13, 16, 29–32]. Furthermore, three cases have been described in the US in individuals who acquired the infection during travel to Italy, Greece, and Africa [33–35]. The question is why does *D. repens* have such a high zoonotic potential in comparison with *D. immitis*?

### *Dirofilaria immitis* vs *Dirofilaria repens*: Why such a difference in the prevalence of human infections?

Currently, there is no evidence indicating the existence of a more virulent *D. immitis* strain in the Americas than in Europe, although recently genetic differences have been observed in worms from human ocular infections both in the Americas and Europe [36, 37], suggesting the existence of a closely related species of *Dirofilaria* [38]. Concerning *D. repens*, while a clear genetic difference has been found among *D. repens*–like worms from Asia (India and Thailand) and between the Asian and the European samples, the genetic variability within the European samples is rather low [39].

One hypothesis to justify such a difference in the zoonotic potential between the two species could be a consequence of an unidentified factor related to the vector. Both *D. immitis* and *D. repens*, however, are able to grow in the same mosquito species and at the same temperature and humidity under laboratory conditions. In addition, both species have the same developmental time from the microfilarial stage to the infective larva [7, 17]. Recently, *D. repens* larvae have been identified in *Anopheles maculipennis* and *Aedes vexans* from Slovakia and Austria [40, 41] and both *D. immitis* and *D. repens* in *Culex pipiens* from Germany and Italy [42, 43]. These mosquito species are well known as competent vectors for *D. immitis* [42]. Therefore, other factors must play a role in the difference in the risk of zoonotic infections between the two species.

In humans, once *D. repens* infective larvae are transmitted by an infected mosquito, in most cases the parasite is found in subcutaneous nodules or in the ocular conjunctiva, apparently near the points of infected mosquito bites [13], although some lung and tumor-like infections have been reported [44–46]. In most cases, the parasite is not able to develop to the sexually mature adult stage and infection is characterized by the presence of pre-adult worms, although at least four cases of microfilaremic zoonotic infections have been reported in Europe and Asia [16, 47]. We can hypothesize that it is much easier for a parasite located in subcutaneous tissues to escape the natural resistance (immune response) of unusual hosts, such as humans, than for *D. immitis* larvae that migrate within deep tissues where they could be killed by the immune system of the host [48].

Most canine *D. repens* infections are subclinical or the clinical signs are nonspecific, and most infections go undiagnosed. Furthermore, while several antigen test kits with high sensitivity and specificity are available for the serologic diagnosis of heartworm infection, no serologic test is available for *D. repens.* This also makes the diagnosis of occult infections, despite clinical suspicion, nearly impossible [49]. It is noteworthy that the lack of cross-reaction between *D. repens* and *D. immitis* [50] is due to the localization of the two parasites (subcutis and bloodstream, respectively) rather than to the specificity of the tests. The heat treatment of serum recently suggested to increase the test sensitivity [51] may increase the risk of cross-reactions between the two *Dirofilaria* species [52]. Examination of the blood for circulating microfilariae is strongly suggested for both *Dirofilaria* infections, but this is the *only* method of *D. repens* diagnosis. Many veterinarians, however, are not familiar with the Knott test, which allows for visualization and identification of microfilariae, particularly those in areas where the parasite has only been recently introduced.

### Prevention and adulticide treatment

The efficacy of the macrocyclic lactones (ML) currently used to prevent *D. immitis* patent infections (ivermectin, milbemycin oxime, moxidectin, and selamectin) was assessed in several experimental laboratory studies and in field studies [3] and all were considered fully effective until the emergence of ML resistance in the United States [53]. No report of resistance has yet been published in Europe. Preventive efficacy against *D. repens* has been evaluated only for the oral ivermectin formulation at 6 μg/kg and 12 μg/kg [54] and both the injectable moxidectin formulation at 0.17 mg/kg and the topical moxidectin formulation at 2.5 mg/kg (in combination with imidacloprid) in experimental studies [10, 55]. Both moxidectin formulations showed full efficacy (100%) while ivermectin showed an efficacy of 87% to 93% [53]. Furthermore, most ML currently used to prevent dirofilarial infections are not fully efficacious against *D. repens* microfilariae; only moxidectin has been approved as a microfilaricidal agent and it must be administered in four monthly doses [56].

Against the adult stages of *D. repens*, the efficacy of melarsomine dihydrochloride currently used against *D. immitis* patent infections is doubtful. There is only one report of an effective treatment obtained with melarsomine plus doramectin in a dog naturally infected with *D. repens* [57], but the data was never confirmed and no efficacy was found in previous clinical studies (Genchi et al., 1996, unpublished). Recently, good adulticidal efficacy (96%) was found in experimentally infected dogs treated monthly for 6 months with moxidectin 2.5 mg/kg body weight (plus imidacloprid 10 mg/kg body weight) [8].

There are several reasons that might explain the difference in efficacy between avermectins and milbemycins. Moxidectin is more lipophilic in nature than ivermectin and it is stored in the fat, which may act as a drug reservoir. Compared with ivermectin, moxidectin has a higher distribution volume and a longer half-life elimination. This may facilitate its distribution from the bloodstream to different tissues and longer residence time for the drug in the body [58, 59]. Furthermore, the recommended dose rate of moxidectin against *D. immitis* infective larvae is 3 μg/kg vs 6 μg/kg of ivermectin [3]. Finally, *D. repens* larval stages and adult worms reside in the subcutaneous tissues, which are rich in fat and connective tissue.

Therefore, the higher lipophilic nature of moxidectin is probably the reason for the full efficacy observed in the studies. Furthermore, P-glycoprotein (P-gp) is a membrane protein belonging to the superfamily of the ATP-binding cassette (ABC) transporters and exerts a potent action vs drug disposition. Moxidectin toxicity depends to a lesser extent on P-gp activity compared with ivermectin (moxidectin seems to be a weaker substrate inhibitor of P-gp than ivermectin [60]). This may explain the higher recommended dose rate of moxidectin vs ivermectin in dogs (eg, 2.5 mg/kg for moxidectin and 6 μg/kg for ivermectin), which therefore makes the spectrum of efficacy of this ML broader.

## Conclusions

For all the reasons discussed in this article, *D. repens* infection is much more difficult than *D. immitis* to diagnose and control in the reservoir population (microfilaremic dogs). If the spread of *D. repens* continues, it is certainly possible that it could be introduced in the United States by translocation of infected dogs. In addition, while veterinarians and pet owners are aware of *D. immitis* infection [61], lack of familiarity with *D. repens* infection could lead to lack of vigilance underestimation for this parasite*.*

The number of human cases in Europe and Asia is currently a serious public health concern. On the other hand, we cannot exclude the possibility that the high zoonotic impact of *D. repens* is not merely a reflection of the more “visible” clinical signs in humans, eg, subcutaneous nodules and conjunctival and subconjunctival infections, which are easier to diagnose than *D*. *immitis* lung infections. Nonetheless, medical doctors and veterinarians must collaborate closely for better control and surveillance of *D. repens* infection. In the Ukraine, reporting cases of dirofilariosis has been mandatory since 1975, and the disease was included in the national surveillance system for notifiable diseases [31]. Guidelines for the control and treatment of *D. repens* infections can be found in the ESCCAP Guideline 5 [62].

## Abbreviations

ESCCAP: European Scientific Counsel Companion Animal Parasites
ML: Macrocyclic lactones
